# The Persistence of Facultative Parthenogenesis in *Drosophila albomicans*

**DOI:** 10.1371/journal.pone.0113275

**Published:** 2014-11-21

**Authors:** Chia-chen Chang, Chau-Ti Ting, Ching-Ho Chang, Shu Fang, Hwei-yu Chang

**Affiliations:** 1 Department of Entomology, National Taiwan University, Taipei, Taiwan 10617, ROC; 2 Department of Life Science, National Taiwan University, Taipei, Taiwan 10617, ROC; 3 Genome and Systems Biology Degree Program, Institute of Ecology and Evolutionary Biology, and Research Center for Developmental Biology and Regeneration Medicine, National Taiwan University, Taipei, Taiwan 10617, ROC; 4 Biodiversity Research Center, Academia Sinica, Taipei, Taiwan 11529, ROC; University of Arkansas, United States of America

## Abstract

Parthenogenesis has evolved independently in more than 10 *Drosophila* species. Most cases are tychoparthenogenesis, which is occasional or accidental parthenogenesis in normally bisexual species with a low hatching rate of eggs produced by virgin females; this form is presumed to be an early stage of parthenogenesis. To address how parthenogenesis and sexual reproduction coexist in *Drosophila* populations, we investigated several reproductive traits, including the fertility, parthenogenetic capability, diploidization mechanisms, and mating propensity of parthenogenetic *D. albomicans*. The fertility of mated parthenogenetic females was significantly higher than that of virgin females. The mated females could still produce parthenogenetic offspring but predominantly produced offspring by sexual reproduction. Both mated parthenogenetic females and their parthenogenetic-sexual descendants were capable of parthenogenesis. The alleles responsible for parthenogenesis can be propagated through both parthenogenesis and sexual reproduction. As diploidy is restored predominantly by gamete duplication, heterozygosity would be very low in parthenogenetic individuals. Hence, genetic variation in parthenogenetic genomes would result from sexual reproduction. The mating propensity of females after more than 20 years of isolation from males was decreased. If mutations reducing mating propensities could occur under male-limited conditions in natural populations, decreased mating propensity might accelerate tychoparthenogenesis through a positive feedback mechanism. This process provides an opportunity for the evolution of obligate parthenogenesis. Therefore, the persistence of facultative parthenogenesis may be an adaptive reproductive strategy in *Drosophila* when a few founders colonize a new niche or when small populations are distributed at the edge of a species' range, consistent with models of geographical parthenogenesis.

## Introduction

Parthenogenesis typically involves females that lay unfertilized eggs that develop into individuals and has independently and recurrently evolved from sexually reproducing ancestors in many multicellular organisms [1], [2]. Most parthenogenetic lineages are facultative, i.e., individuals can reproduce by both parthenogenesis and sexual reproduction [3]–[7], whereas very few lineages, such as bdelloid rotifers, have been successful for millions of years under obligate parthenogenesis, in which individuals are exclusively parthenogenetic [8]. In the latter case, however, alternate genetic mechanisms that facilitate long-term persistence involving DNA damage and repair are involved [9].

Parthenogenesis has been reported in more than 10 *Drosophila* species [10]–[19]. All of the species with the exception of *D. mangabeirai* exhibit tychoparthenogenesis, which is occasional or accidental parthenogenesis in a normally bisexual species with a low hatching rate of eggs produced by virgin females and is presumed to be the first step in the evolution of parthenogenesis [1]. As in some *Drosophila* [13], [18], [19], cases are classified as facultative parthenogenesis in a broad sense; thus, we use both tychoparthenogenesis and facultative parthenogenesis interchangeably to refer to parthenogenesis in *Drosophila*. Several important reproductive features that are seen during the early stage of parthenogenesis contribute to the evolutionary fate of facultative parthenogenesis in *Drosophila*. The first such feature is mating propensity or the probability of mating with males. Mating propensity might not be a necessary criterion for some types of parthenogenesis, such as gynogenesis and hybridogenesis, in which mating with males is required although no paternal genetic material is contributed to the offspring. However, such parthenogenetic forms have not been found in *Drosophila* [20]. In several independent parthenogenetic strains of *D. mercatorum*, mating propensity decreased after isolation from males [21]. The mating propensities of parthenogenetic females decreased to 80–85% after 10 years of isolation and were further reduced to less than 35% after 20 years of isolation, whereas the mating propensities of their parental sexual strains remained at approximately 85–90%. Schwander et al. [22] described a model in which tychoparthenogenesis under mate limitation in natural populations could evolve to obligate parthenogenesis through a positive feedback mechanism. If mutations reducing mating propensity also occur under mate limitation in natural populations, decreased mating propensity might accelerate the feedback process. The second feature addresses fertility, which determines the evolutionary success of parthenogenetic lineages. If the fertility of parthenogenetic and sexually reproducing females were the same, parthenogenesis would have an advantage over sexual reproduction, as the latter represents a “two-fold cost of sex,” in which sexually reproducing females waste half of their reproductive potential on producing male progeny [23]. However, parthenogenetic *Drosophila* often exhibit lower fertility than their sexually reproducing counterparts [10], [11], [13]. The third feature is parthenogenetic capability — the ability of unfertilized eggs to complete development. In facultatively parthenogenetic *Drosophila*, the parthenogenetic capability of individuals can be preserved in mated parthenogenetic females and their sexual offspring for many generations [13], [15], [24]–[26]. Therefore, facultative parthenogenesis can persist in the population using two alternative reproductive modes depending on the availability of males. The fourth feature is how diploidy is restored in unfertilized eggs. In successful parthenogenetic lineages, such as bdelloid rotifers and *Timema* stick insects, unfertilized diploid eggs are formed by apomixis without meiosis [8], [22], whereas most low-fertility parthenogenetic lineages restore diploidy by automixis, which is meiosis followed by diploidization. The fertility reduction in automictically parthenogenetic lineages, such as *D. mercatorum*, may be related to the high failure rate of diploidy restoration due to the low success rate of centrosome formation without the paternal basal body [27], [28]. The importance of centrosomes has also been demonstrated; *Wolbachia*-induced parthenogenesis occurs more easily in haplodiploid hymenopterans, in which haploid males are pre-adapted to inherit maternal centrosomes [29]–[31]. Diploidization usually occurs through three main cytological mechanisms: gamete duplication, in which chromosome doubling occurs after meiosis II; the central fusion of two nuclei derived from different meiosis I nuclei; and the terminal fusion of two nuclei derived from the same meiosis I nucleus [3]–[7]. Both central fusion and terminal fusion can maintain a portion of heterozygosity, whereas gamete duplication enforces homozygosity at all loci. If central fusion with very low genetic recombination is a major mechanism of diploidization, parthenogenesis may retain adaptive heterozygous genotypes in the population [7], [32], [33]. The fact that the only obligately parthenogenetic *Drosophila* species, *D. mangabeirai*, exclusively uses central fusion and exhibits permanent heterozygosity in two chromosomal inversions [11], [34], [35] might reflect the importance of central fusion with crossover suppressors for obligate parthenogenesis. By contrast, if gamete duplication is the major mechanism, such as in the well-studied facultatively parthenogenetic cases of *D. mercatorum* [13], and *D. ananassae* complex species [15], [25], [36], the genetic variation in parthenogenetic individuals would be very low. Accordingly, these four reproductive features might explain why many *Drosophila* parthenogenetic lineages are facultative rather than obligate.

Although reproductive features in tychoparthenogenetic *Drosophila* species have been studied for more than 60 years, the genetics underlying the parthenogenetic system is largely unknown. With a long history of evolutionary studies [37] and a reference genome [38], *D. albomicans* provides a good model for the genetic study of tychoparthenogenesis. Here, we characterize the reproductive features of tychoparthenogenetic *D. albomicans* to understand the early stage of the evolution of parthenogenesis. First, the mating propensity of the parthenogenetic strain after 20 years of culture without males was investigated. Second, the fertility of this parthenogenetic strain was measured and the reproductive patterns of mated parthenogenetic females were assessed. Third, parthenogenetic capability was assessed by measuring both the hatchability of unfertilized eggs and the percentage of females capable of parthenogenesis. Fourth, diploidization mechanisms were characterized to assess the genetic variation of parthenogenesis in *D. albomicans*. Finally, the potential evolutionary fates of tychoparthenogenesis in *Drosophila* are discussed.

## Materials and Methods

### Fly strains

The parthenogenetic *D. albomicans* strain was derived from an isofemale line, KKU119, collected from Kiikatsuura, Japan, in 1990 [18]. To generate a parthenogenetic strain with a visible marker, KKU119 females were crossed with males of a sexual strain with a spontaneous recessive eye color mutation, *bordeaux* (*bo*), a generous gift from Stéphane Prigent. After 4–5 generations of free recombination between these two strains, 34 parthenogenetic lines with bordeaux eye color were selected. An additional 15 molecular markers were genotyped to estimate the proportion of introgression in these different KKU119-bo lines. The line with the lowest introgression (12.5%, one out of 15 molecular markers and one eye-color marker) was selected for this experiment. The two sexual strains (percentage of virgin females capable of producing offspring  =  0%, see Results) used for comparison were #55.1, an isofemale line established in 1970 from Hualien, Taiwan, and #163.5-IL, an inbred line originally collected in 1978 from Okinawa, Japan. Flies were maintained on standard cornmeal medium at 23±1°C under a 12∶12 LD cycle. Virgins were collected within 8 hours after eclosion.

### Mating propensity

The mating propensities of 4-day-old virgin KKU119 females were assayed by placing individual females in a vial with a 4-day-old #55.1 male. Mating propensity was measured as the percentage of pairs that copulated within two hours. For comparison, the mating propensities of #55.1 X #55.1 and #163.5-IL X #55.1 were also assessed. The hatchability of virgin KKU119 females was calculated by counting the number of hatched larvae from 100 eggs.

### Fertility

The fertility of parthenogenetic KKU119 females was estimated by the average number of offspring produced by ∼3- to 4-day-old individuals. The fertility of mated KKU119 females was estimated by the average number of offspring produced by a 4-day-old female paired with a 4-day-old #55.1 male for four weeks. As a comparison, the fertility of single-pair #55.1 flies was also determined. The offspring of the cross between KKU119-bo females (*bo*/*bo*) and #55.1 (+/+) were examined to quantify the number of offspring produced by parthenogenesis (*bo*/*bo*, bordeaux eye color females) and by sexual reproduction (*bo*/*+*, wild-type eye color females and males). To examine the effect of mating times on these two reproductive modes, we divided cross groups as (A) a single mating group in which a single pair mated within two hours and the male was removed from the vial after mating and (B) a potential multiple-mating group in which a single pair mated within two hours and both the male and female were kept in the vial after mating to allow for possible multiple mating. In all experiments, single virgin females or single-pair crosses were placed in a vial and transferred twice per week for four weeks. The numbers of offspring produced in each week and in four weeks were defined as weekly and total fertility, respectively. Females that died within four weeks or produced no offspring were excluded from further analysis.

### Parthenogenetic capability

Parthenogenetic capability was measured as the hatchability of eggs produced by virgin KKU119 females. Eggs from #55.1 X #55.1 matings were also examined for hatchability for comparison. In addition, the percentage of virgin females capable of producing offspring was measured. KKU119 females and their F_1_ progeny produced by crosses with sexual strain males, i.e., KKU119 X #55.1 and KKU119 X #163.5-IL, were assayed for parthenogenetic capability. For comparison, #55.1 and #163.5-IL females were also assayed. Parthenogenetic capability was calculated as the percentage of tested virgin females that produced offspring within four weeks. Virgin females were placed individually in vials and transferred to a new vial twice per week for four weeks. Because mated KKU119 females could produce offspring by both parthenogenesis and sexual reproduction (see Results), the F_1_ females of KKU119 X #55.1 and KKU119 X #163.5-IL were genotyped with at least one molecular marker, and only heterozygous F_1_ progeny, indicating parthenogenetic-sexual hybrids, were used.

### Determination of diploidization mechanisms

**Figure 1:**
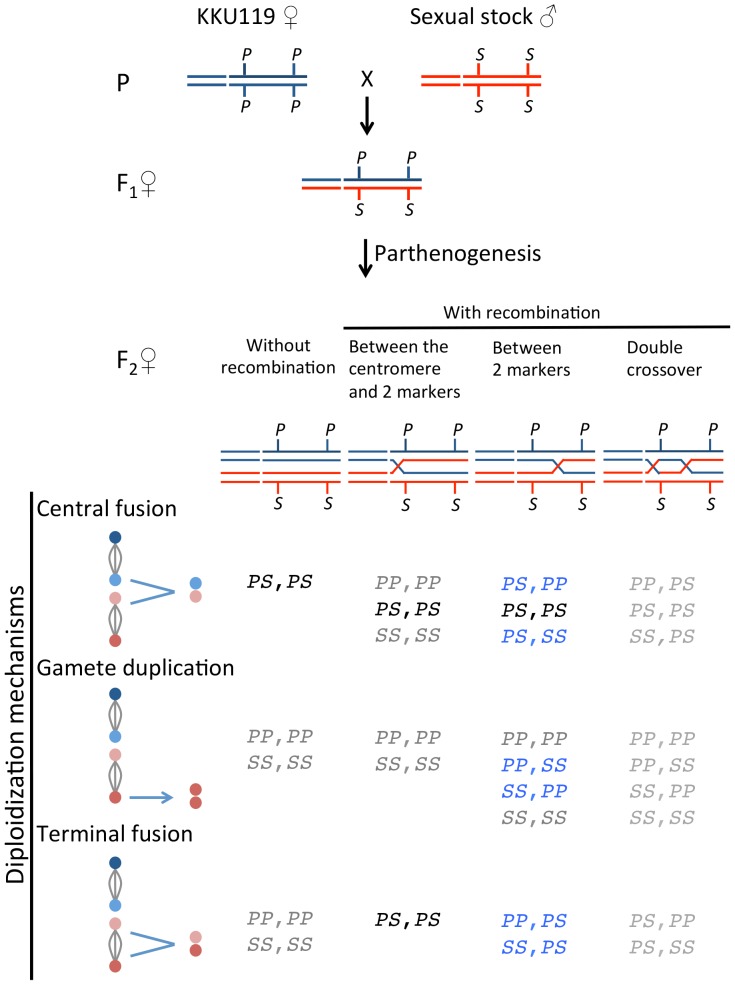
Possible genotypes based on different diploidization mechanisms in parthenogenesis in *Drosophila albomicans*. The three possible diploidization mechanisms are illustrated in the left column. The four nuclei produced after meiosis are indicated by colored circles. Central fusion is the fusion of two nuclei derived from different meiosis I nuclei (“dark blue fusion with pink or red circles” or “light blue fusion with pink or red circles”), whereas terminal fusion is derived from the same meiosis I nucleus (“dark blue fusion with light blue circles” or “pink fusion with red circles”). Gamete duplication is the fusion of two nuclei duplicated from one of four nuclei. All possible genotypes of two linked markers are listed under three mechanisms with (A) no recombination, (B) one recombination event between two markers and the centromere, (C) one recombination event between two markers, and (D) double crossover. The chromosomes from parthenogenetic and sexual strains are labeled by blue and red, respectively. The alleles of each locus are labeled as *P* for parthenogenetic strain and *S* for the two sexual strains. Because the double-crossover rate was extremely low, recombinant F_2_ offspring arising from double crossover are not considered in this analysis. Genotypes that can have come from only one of the three mechanisms are labeled in blue, the genotypes in black (i.e., *PS*,*PS*) are from central fusion or terminal fusion, and genotypes that are shared by the three mechanisms are labeled in gray.

To infer the diploidization mechanism of parthenogenesis, females that are heterozygous at multiple loci are required. Because there were very few heterozygous loci in KKU119, heterozygous females were obtained from the F_1_ progeny of the crosses between parthenogenetic strain females and sexual males. All parthenogenetic-sexual F_1_ hybrid females were confirmed by genotyping. Diploidization mechanisms were determined by multiple- and two-marker methods. We first used 13 markers spread across two major chromosomes to distinguish gamete duplication from fusions in the F_1_ hybrid females of KKU119 X #55.1 (Additional file 1 of [39], but the a52 marker and one individual case of the c29 marker with missing data were excluded from analyses). An F_2_ individual with any marker heterozygous must be produced by either central fusion or terminal fusion because gamete duplication would enforce all loci homozygous. To further distinguish central fusion events from terminal fusion events, we used two markers on the same chromosomal arm to infer different diploidization mechanisms. This analysis was performed in the F_1_ hybrids of the crosses of both KKU119 X #55.1 and KKU119 X #163.5-IL. Based on the predicted genotypes (Figure 1), recombinant F_2_ progeny provided better resolution than non-recombinant F_2_ progeny in distinguishing the three possible diploidization mechanisms. As recombination rates were crucial, two markers with a genetic distance greater than 50 cM on the neo-X chromosome (a1350: near the centromere and c29: near the telomere [37]) were used. Alleles from parthenogenetic and sexual strains were designated as *P* and *S*, respectively. Because the a1350 marker was very close to the centromere, double-crossover events, one between the centromere and a1350 and the other between a1350 and c29, were too low to be considered in this analysis. Among the nine possible genotypes, *PP*,*SS* and *SS*,*PP* were uniquely produced by gamete duplication. Genotypes *PS*,*PP* and *PS*,*SS* were most likely produced by central fusion, whereas genotypes *PP*,*PS* and *SS*,*PS* were most likely produced by terminal fusion. The doubly heterozygous genotype (*PS*,*PS*) could be produced either by central fusion (with or without recombination) or by terminal fusion with a crossing over between a1350 and the centromere.

### Genotyping

Single-fly genomic DNA was extracted using the Gentra Puregene Cell and Tissue DNA Isolation Kit (Qiagen, the Netherlands) following the manufacturer's instructions. The PCR-RFLP markers on the neo-X chromosome arm were used to distinguish alleles between parthenogenetic and sexual strains. The chromosomal location, primer sequences, and PCR condition for each marker were described in [39].

## Results

### The long-term-isolated parthenogenetic strain without males had a low mating propensity

To examine whether the females of parthenogenetic strain KKU119 had reduced ability to reproduce sexually after long-term (> 20 years) laboratory culture without males, we first measured their mating propensity. Among 20 single pairings of KKU119 females with #55.1 males, only 35.0% of KKU119 females mated within two hours, whereas both of the sexual strains had significantly higher mating propensities (Table 1). Thus, mating propensity was reduced after 20 years of parthenogenesis.

**Table 1:** Female mating propensities of the parthenogenetic and sexual strains of *Drosophila albomicans.*

		Proportion of pairs that mated within two hours	
Strain^a^	Sample size	%	Probability of no difference between KKU119 and sexual strains by Fisher's exact test
KKU119	20	35.0	
#55.1	19	84.2	*P* = 0.03
#163.5-IL	14	93.3	*P*<0.001

a. One female from each strain was paired with one male from #55.1.

### The parthenogenetic strain had lower fertility

To assess fertility differences between parthenogenetic and sexually reproducing strains, we measured the total numbers of offspring of virgin KKU119, mated KKU119 (KKU119 X #55.1), and mated #55.1 (#55.1 X #55.1) females for 4 weeks. The total 4-week fertility of virgin KKU119 females was significantly lower than that of mated KKU119 females (43.0±14.4 vs. 182.9±106.0, *t* = −7.63, *df* = 73, *P*<0.0001). The total fertility of mated KKU119 females was lower than that of mated #55.1 females (319.8 ± 100.3; *t* = −4.64, *df*  =  57, *P* <0.0001).

**Figure 2:**
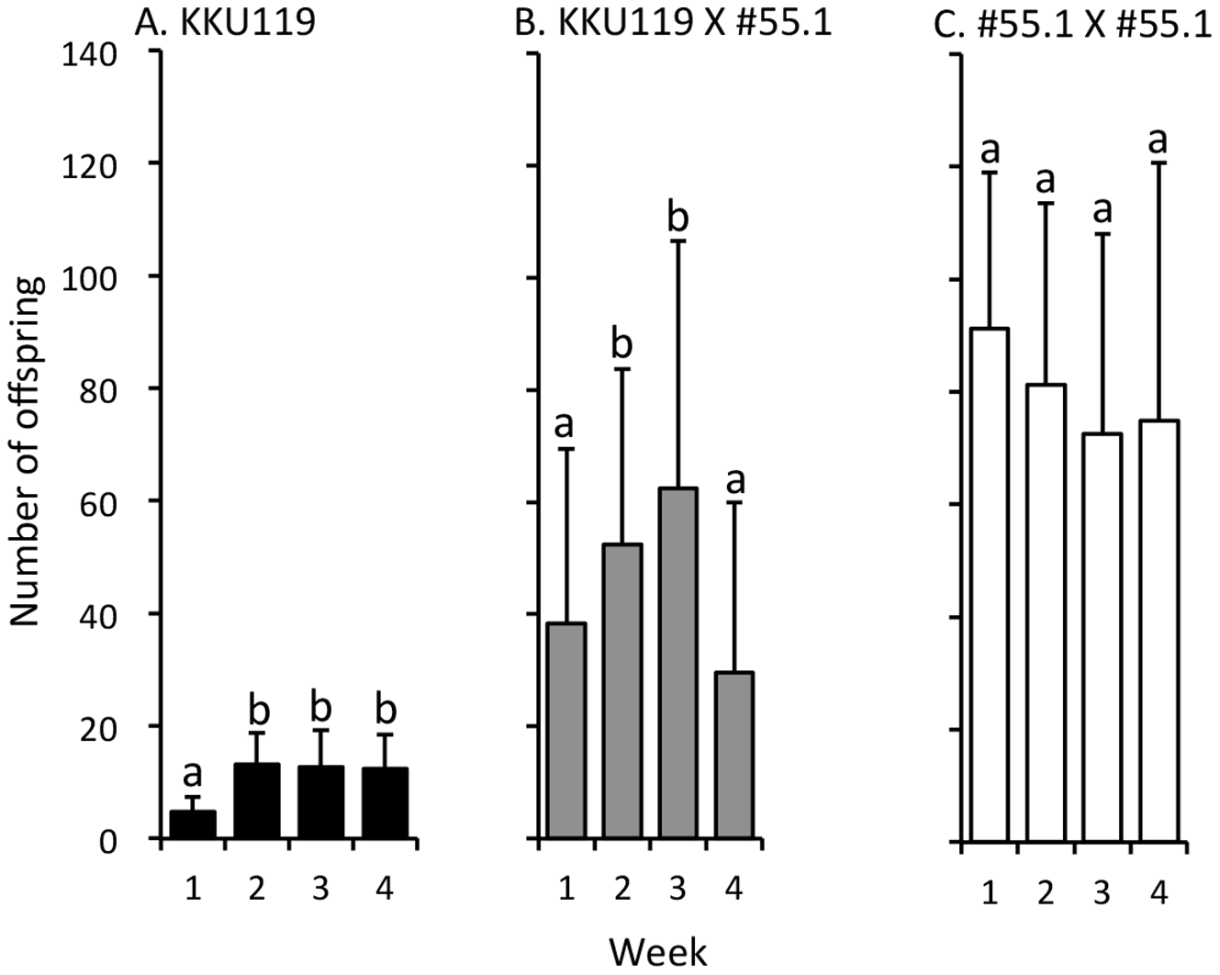
Weekly offspring numbers of single virgin KKU119 (A), mated KKU119 (B), and mated #55.1 (C) females of *Drosophila albomicans*. The X-axis denotes weeks after mating. The Y-axis denotes offspring numbers. Sample size: A  =  34, B  =  41, C  =  18. Error bars indicate standard errors. Different letters indicate that the values are significantly different between weeks at *P*<0.05 by Friedman's test and an additional post hoc analysis of Bonferroni-corrected Wilcoxon signed-rank test.

The weekly fertility patterns varied among virgin KKU119, mated KKU119, and mated #55.1 females. In virgin KKU119 females, fertility was lower in Week 1 but increased to similar levels in the last three weeks (Figure 2A). In mated KKU119 females, fertility was lower in Week 1, increased in Weeks 2 and 3, and then decreased in Week 4 (Figure 2B). In mated #55.1 females, fertility did not change over time (Figure 2C).

### Mated KKU119 females produced progeny predominantly by sexual reproduction

**Figure 3:**
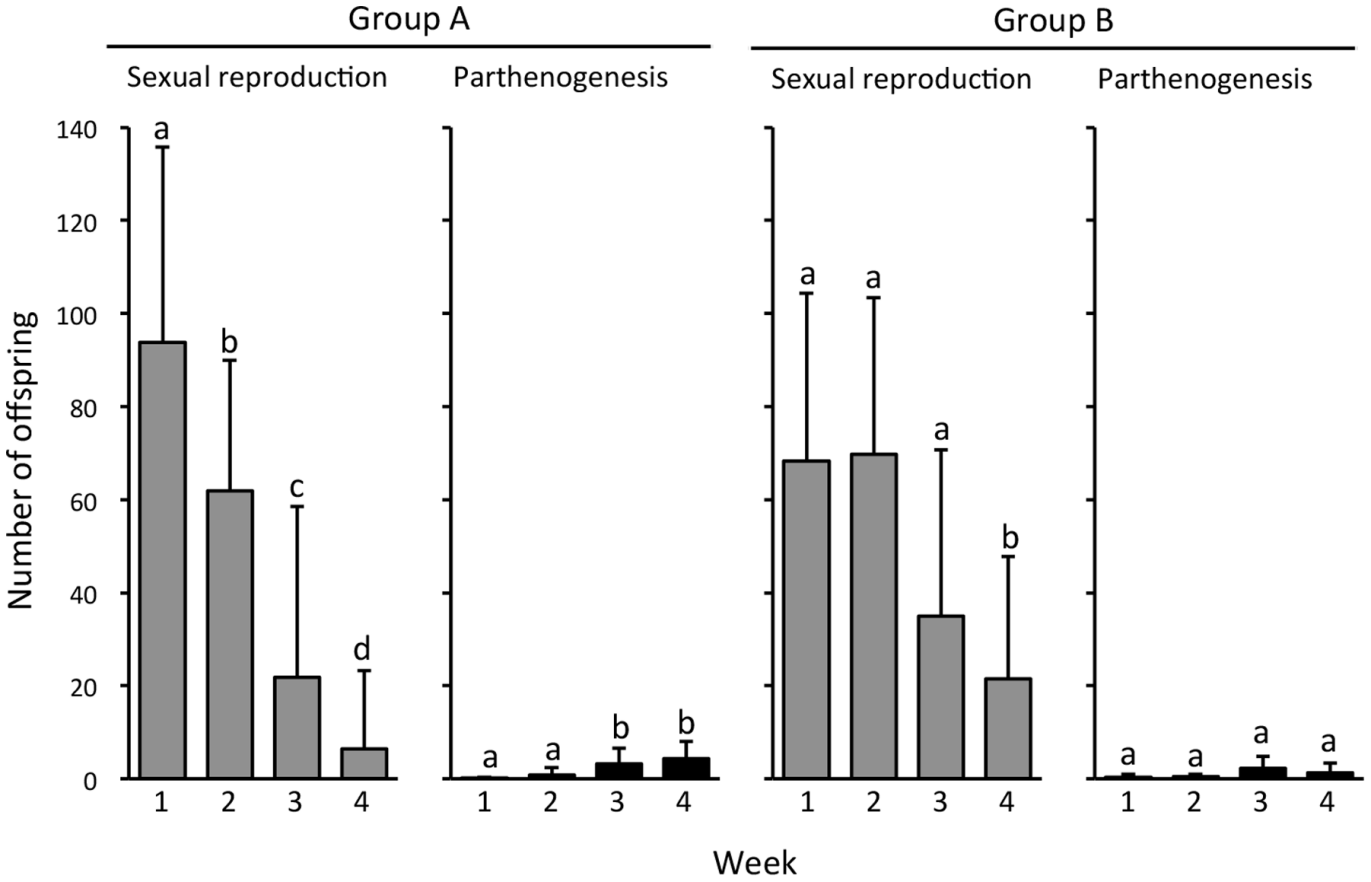
Weekly offspring numbers of mated KKU119-bo females. Group A: the single mating group in which single pairs mated within 2 hours and the male was removed from the vial. Group B: the potential multiple mating group in which after mating, the male was kept in the vial to allow potential multiple mating. Error bars indicate standard errors. Different letters indicate that offspring numbers are significantly different between weeks at *P*<0.05 by Friedman's test and an additional post hoc analysis of Bonferroni-corrected Wilcoxon signed-rank test.

**Figure 4:**
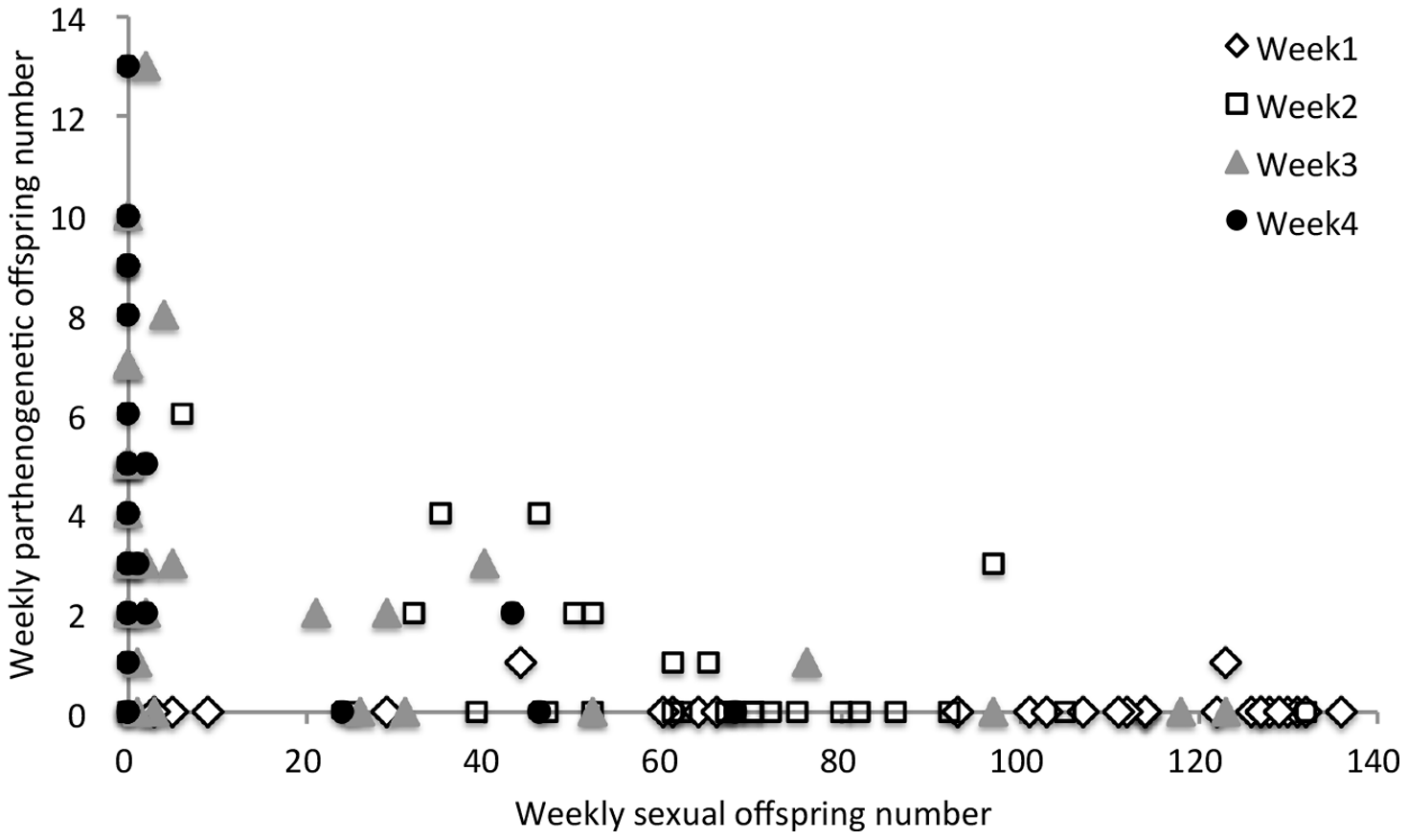
A negative correlation between the weekly offspring numbers by parthenogenesis and by sexual reproduction in mated KKU119-bo females of the single mating group. Spearman's *ρ* = −0.58, *P* = 4.99×10^−10^.

To investigate whether mated facultatively parthenogenetic females could produce progeny by parthenogenesis, we crossed 70 single females of KKU119-bo to single males of sexual strain #55.1 and counted the numbers of their progeny produced by parthenogenesis and by sexual reproduction (see Materials and Methods). As the number of mating events might affect the quantities of functional sperm and seminal fluid proteins and thus influence the number of sexual offspring, we divided the 46 females that mated within two hours (mating propensity = 65.7%) into a single mating group (group A) and a potential multiple-mating group (group B), which differed by whether the male was kept in the vial after mating. Except for one female in group A without offspring, almost all females produced male offspring and female offspring with two eye color phenotypes, indicating that mated KKU119-bo produced offspring by both sexual reproduction and parthenogenesis. In both groups, mated KKU119-bo females produced progeny predominantly by sexual reproduction (Figure 3). As expected, mating events affected the number of sexual offspring and the profile of parthenogenetic offspring production. The number of sexual offspring was significantly reduced over time in group A but only reduced at the fourth week in group B (Figure 3). The weekly patterns of parthenogenetic offspring numbers between groups A and B were also different. A significant difference in parthenogenetic offspring numbers was detected among weeks in group A but not in group B. In group A, more parthenogenetic offspring were produced in Weeks 3 and 4 than in the first two weeks (Figure 3). Furthermore, a significant negative correlation between weekly offspring numbers produced by sexual reproduction and by parthenogenesis was observed in group A (Figure 4). Therefore, it was likely that the increase in the number of parthenogenetic offspring in the last two weeks of group A was due to a shortage of sperm and/or seminal fluid proteins transferred from only one mating and thus reduced production of offspring by sexual reproduction in the last two weeks (Figure 4). Especially in Week 4, 75.9% of females produced no sexual offspring. Total numbers of parthenogenetic offspring in these two groups were also different. The average total number of parthenogenetic offspring was significantly higher in group A (8.6±6.3, N = 29) than in group B (4.3±4.2, N = 11) (*t* = 2.09, *df* = 38, *P* = 0.043), but both were dramatically lower than in virgin KKU119-bo females (15.2±7.4, N = 10) (Mann-Whitney *U* test; group A vs. virgin KKU119-bo, *P* = 0.0226; group B vs. virgin KKU119-bo, *P* = 0.0035).

KKU119-bo flies might be different from the parthenogenetic strain in predominantly producing sexual offspring from mated females because in KKU119-bo, introgression from a sexual strain might increase fertilization rates and thus increase the proportion of sexual offspring. However, this possibility can be ruled out because the results of molecular genotyping showed that a small fraction of offspring produced by mated KKU119 (data used in Figure 2B) were similar to those of KKU119-bo with regard to both the predominant production of sexual offspring and the negative correlation between the numbers of sexual offspring and parthenogenetic offspring produced in each week (Spearman's *ρ* = −0.66, *P* = 0.0039). This finding suggests that a small amount of introgression from the sexual strain did not affect the ability to produce sexual offspring after mating.

### F_1_ female progeny of sexual reproduction by the parthenogenetic strain had the capability to perform parthenogenesis but produced very few progeny

The parthenogenetic capability estimated by the hatchability of eggs produced by virgin KKU119 females was 23% (N = 100 eggs), which was much lower than that of mated #55.1 females (88%, N = 100 eggs). All the virgin KKU119 females that were tested produced progeny parthenogenetically in four weeks (Table 2). F_1_ females produced by sexual reproduction from crosses between KKU119 and the sexual strains retained their ability to reproduce parthenogenetically, but this ability was decreased compared with that of KKU119 (75.3% for F_1_ from #55.1 cross set, χ^2^ = 10.48, *P*<0.01; 69.0% for F_1_ from #163.5-IL cross set, χ^2^ = 13.62, *P*<0.001). In addition, for both crosses, each F_1_ female on average produced fewer than two offspring, which was much lower than the number produced by the parthenogenetic strain females (Mann-Whitney *U* test, *P*<0.001; Table 2).

**Table 2:** The percentage of virgin females capable of producing progeny and the fertility of virgin females from three strains of *Drosophila albomicans* and their sexually produced F_1_ progeny.

Virgin female	Sample size	Percentage of virgin females capable of producing progeny (%)	No. offspring (Mean ± SE)
KKU119	34	100	43.0±14.4
F_1_ from KKU119♀ x #55.1♂	146	75.3	1.4±1.4
F_1_ from KKU119♀ x #163.5-IL♂	113	69.0	1.1±1.1
#55.1	72	0	0
#163.5-IL	16	0	0

### Gamete duplication was the predominant diploidization mechanism

To assess the diploidization mechanisms in the parthenogenetic system of *D. albomicans*, we first used 13 markers to distinguish gamete duplication from fusions. The 13-marker data from the cross of KKU119 X #55.1 showed that 15.2% of F_2_ progeny (N = 104) were heterozygous for at least one marker and thus formed via fusion. The proportion of F_2_ females produced by fusion is a minimum estimation, and therefore, the proportion of gamete duplication was less than 85.6%. To distinguish central fusion from terminal fusion, we applied the method shown in Figure 1 using two markers with a large genetic distance for the KKU119 X #55.1 cross set and another cross set, KKU119 X #163.5-IL. The KKU119 X #55.1 cross set showed that 50 out of 104 F_2_ progeny were distinguishable for different diploidization mechanisms; 94.0% by gamete duplication, 0% by central fusion, and 6.0% by terminal fusion. The #163.5-IL cross set showed that 47 out of 103 F_2_ progeny were distinguishable; 89.4% by gamete duplication, 2.1% by central fusion, 6.4% by terminal duplications, and 2.1% by either central fusion or terminal fusion (Table 3). The two cross sets did not have significantly different proportions of gamete duplication (Fisher's exact test, *P* = 0.75). Although the two-marker method overestimated the proportion of gamete duplication, both methods revealed that the predominant mechanism was gamete duplication. Multiple diploidization mechanisms can occur in single females; of the 43 F_1_ females that yielded more than one offspring, five showed clear evidence of both gamete duplication and fusion mechanisms (Table S1).

**Table 3:** Percentages of various diploidization mechanisms of parthenogenesis in *Drosophila albomicans.*

	Datasets from F_1_ of crosses between KKU119 and two sexual strains		
Diploidization mechanism (genotypes)*	KKU119 X #55.1	KKU119 X #163.5-IL	Sum
Gamete duplication (*PP*,*SS* and *SS*,*PP*)	47 (94.0%)	42 (89.4%)	89 (91.8%)
Fusion			
Central fusion (*PS*,*PP* and *PS*,*SS*)	0 (0%)	1 (2.1%)	1 (1.0%)
Terminal fusion (*PP*,*PS* and *SS*,*PS*)	3 (6.0%)	3 (6.4%)	6 (6.2%)
Central or terminal fusion (*PS*,*PS*)	0 (0%)	1 (2.1%)	1 (1.0%)

* See Figure 1 for the inference of diploidization mechanisms by different genotypes.

## Discussion

As in other parthenogenetic *Drosophila* species [21], long-term (20 years, ∼370 generations) isolation from males resulted in a reduced mating propensity in parthenogenetic *D. albomicans* females. The fertility of mated parthenogenetic females was significantly higher than that of virgin females. The mated parthenogenetic females produced offspring partly by parthenogenesis but predominantly by sexual reproduction. This predominance of sexual reproduction might be common in *Drosophila* species because a similar phenomenon has also been found in *D. mercatorum* [14]. The parthenogenetic capability can also be preserved in parthenogenetic-sexual hybrids. As in *D. ananassae*-complex species [15], [25], [36] and *D. mercatorum* [13], [24], gamete duplication is the major diploidization mechanism. These reproductive features suggest that *D. albomicans* is a typical tychoparthenogenetic *Drosophila* species.

### The low fertility of parthenogenetic females may be attributable to low fecundity and low hatchability

At least two fitness components, fecundity and hatchability, might account for the low fertility in the parthenogenetic strain of *D. albomicans*. Virgin females in both KKU119 and #55.1 laid a lower number of eggs than did mated females (Table S2). The low fecundity of these virgin females might be partly due to the lack of sperm and seminal fluid proteins (e.g., Acp26Aa and CG33943), which stimulate females to lay eggs after mating in *Drosophila* [40], [41]. By forcing parthenogenetic females to mate only once (i.e., single mating group) to limit the amount of sperm and seminal fluid proteins transferred, we found that the number of offspring by sexual reproduction decreased over weeks, whereas the number of offspring by parthenogenesis increased in the last two of the four experimental weeks. This pattern was not observed in the potential multiple-mating group, in which the proportions of parthenogenetic offspring did not vary over the four-week period.

Eggs laid by KKU119 virgin females had lower hatchability than those laid by mated #55.1 females. The lower egg hatchability may be similar to the case of *D. mercatorum*, in which only a small proportion of unfertilized eggs exhibited the successful *de novo* formation of functional centrosomes to circumvent the deficit of the sperm-provided centrosomes to develop into diploid individuals [27], [28].

### No evidence for parthenogenesis induced by *Wolbachia*

*Wolbachia*-induced parthenogenesis has been well studied in haplodiploid hymenopterans, thrips, and mites but has not been described in *Drosophila* [43]. Two lines of evidence suggest that parthenogenesis in *D. albomicans* is unrelated to *Wolbachia*. First, one virgin F_1_ female from the cross between the KKU119 females and the males of the two sexual strains produced only one progeny on average vs. the many offspring produced by their KKU119 mothers. This result implies that the parthenogenesis of *D. albomicans* might not be *Wolbachia* induced. Otherwise, the cytoplasmically inherited *Wolbachia* would be transmitted to F_1_ females to induce parthenogenesis with many offspring. Second, by using a pair of universal primers to detect the 16S rRNA gene of *Wolbachia* [42], our preliminary result showed no sign of infection by *Wolbachia* in the parthenogenetic *D. albomicans* strains (Figure S1). Two pre-adapted features make the haplodiploid sex-determination system successful for the transition to obligate parthenogenesis [43]. First, haplodiploid hymenopterans have evolved cytoplasmic organelles called accessory nuclei to facilitate the formation of maternal centrosomes [44], [45]. These maternal centrosomes play an important role in the embryogenesis of unfertilized eggs of parthenogenetic haplodiploid hymenopterans. In contrast, most unfertilized eggs in parthenogenetic *Drosophila* fail to develop due to the lack of accessory nuclei to form maternal centrosomes [27], [28]. Second, haploid males have acted as a sieve against recessive deleterious mutations, and therefore, haplodiploid species are more tolerant of inbreeding resulting from the increased homozygosity by gamete duplication than are diploid species such as *Drosophila* [43], [46].

### The evolutionary fate of facultative parthenogenesis

Tychoparthenogenesis has evolved independently multiple times in *Drosophila*, but only one obligate species has been reported [19]. It has been proposed that tychoparthenogenesis is the first step in the evolution of obligate parthenogenesis [1]. If we focus only on the fertility differences revealed in this study by considering the two-fold cost of sex [23], the fitness of the parthenogenetic strain is too low to compete with sexual strains, as reported in most parthenogenetic cases [1], [6]. It is therefore likely that any accidentally parthenogenetic lineage in *Drosophila* would go extinct.

The second possible fate of tychoparthenogenesis is obligate parthenogenesis. Using *Timema* stick insects as a model, Schwander et al. [47] demonstrated that if females are mate limited, tychoparthenogenesis can generate female-biased sex ratios and increasing mate limitations and thus result in the loss of males through a positive feedback mechanism. Our data showed that the mating propensity of a facultative strain in *D. albomicans* was reduced to 35% after a 20-year laboratory culture without males. If mutations that reduce mating propensities could occur under mate limitation in natural populations, decreased mating propensity might accelerate the positive feedback loop. This process provides an opportunity for the evolution of obligate parthenogenesis.

The next question is whether parthenogenetic capability is heritable. If it is, does genetic variation in parthenogenetic capability exist in natural populations? Our data showed that parthenogenetic-sexual hybrid offspring in *D. albomicans* can produce offspring parthenogenetically, indicating that parthenogenetic capability is a heritable trait. The parthenogenetic strain KKU119 was established from an isofemale stock by artificial selection [18]. As in domesticated animals and plants, artificial selection acts on natural heritable variation [48]; thus, genetic variation in loci that contribute to parthenogenesis must pre-exist in natural populations. Supporting evidence comes from an independent observation that females from KKU204, another isofemale strain from Kiikatsuura, Japan, in 1991, could also perform parthenogenesis (Kazuhiro Satomura, pers. comm., [39]), and their parthenogenetic offspring could reproduce parthenogenetically (Shu Fang, unpublished data). The fact that another Kiikatsuura line also can reproduce parthenogenetically suggests that the parthenogenetic alleles of *D. albomicans* do exist in nature, at least in the Kiikatsuura population.

Diploidization mechanisms are also an important factor affecting the evolutionary fate of tychoparthenogenesis. Central fusion coupled with partial or complete crossover suppression preserves a higher level of heterozygosity and has been suggested as the most common type in obligately automictic species such as *D. mangabeirai* [49]. Although gamete duplication is the predominant mechanism of diploidization in *D. albomicans*, central fusion and terminal fusion are also observed. The genetic polymorphism in diploidization mechanisms provides potential for central fusion, in combination with crossover suppressors, to become the predominant mechanism. It is possible that parthenogenetic females that adopt central fusion will be selected to form an obligate parthenogenetic species.

The third possible fate of tychoparthenogenesis is coexistence with sexual reproduction. Genetic variation in reproductive features plays an important role in the evolutionary success of parthenogenesis in *D. albomicans*. If we only consider that parthenogens are mainly produced by gamete duplication, it is difficult for tychoparthenogenesis to persist in natural populations due to limited genetic variation. However, these parthenogens are capable of sexual reproduction when males are available. Genetic variation in facultative parthenogens can be elevated by sexual reproduction. After mating, parthenogenetic females preferentially produce offspring by sexual reproduction. Although parthenogenetic-sexual hybrid females, such as F_1_ females, might suffer low parthenogenetic fertility, the alleles responsible for parthenogenesis are propagated in these hybrids and their descendants in natural populations, such as the Kiikatsuura population. Individuals capable of parthenogenesis are presumably adaptive, as finding mates is difficult in small populations, such as founders that colonize isolated areas or populations that are distributed at the boundaries of the species' range. This phenomenon has been documented in various parthenogenetic organisms [6], [7], [50], [51] and is also the case for *D. albomicans*. The female used for the establishment of the parthenogenetic *D. albomicans* strain was collected at the edge of the geographic distribution of the species [18], [52], where it may be difficult for females to find males after cold winters. Taken together, these aspects of parthenogenesis might explain the coexistence of parthenogenesis and sexual reproduction in *D. albomicans*.

In summary, we have demonstrated that the capacity to reproduce parthenogenetically is a heritable trait and that the alleles responsible for parthenogenesis can be maintained through both parthenogenesis and sexual reproduction. Parthenogenesis is advantageous when females are mate limited, whereas sexual reproduction can introduce genetic variation into the parthenogenetic genome. These results suggest that facultative parthenogenesis is an adaptive reproductive strategy and might evolve further to obligate parthenogenesis in suitable geographic, ecological, and genetic circumstances [49].

## Supporting Information

Figure S1 No evidence for *Wolbachia* infection in the parthenogenetic KKU119 of *Drosophila albomicans. Wolbachia*-specific primers, *W-Specf* and *W-Specr* [a], were used to amplify the *16S rRNA* gene. To ensure the DNA quality, the primers, *tLEU* and *tLYS* [b], were used to amplify the mitochondrial *COII* gene. The *D. ananassae* strain 14024-0371.13 containing *Wolbachia* nuclear insert [c] was used as the positive control. Different numbers denote different KKU119 females. M: 100 bp DNA ladder. PC: positive control. NC: negative control. a. Werren JH, Windsor DM (2000) Wolbachia infection frequencies in insects: evidence of a global equilibrium? Proc R Sci Lond B 267: 1277-1285. b. Simon C, Frati F, Beckenbach A, Crespi B, Liu H, Floors P (1994) Evolution, weighting, and phylogenetic utility of mitochondrial gene sequences and a compilation of conserved polymerase chain reaction primers. Ann Entomol Soc Am 87: 651-701. c. Dunning Hotopp JC, Clark ME, Oliveira DCSG, Foster JM, Fischer P, et al. (2007) Widespread lateral gene transfer from intracellular bacteria to multicellular eukaryotes. Science 1753-1756.
(DOCX)

Table S1 Diploidization mechanisms of F_1_ female *Drosophila albomicans* with more than one offspring.
(DOCX)

Table S2 The average cumulative number of eggs laid by 4-day-old virgin and mated females of *Drosophila albomicans*.
(DOCX)
