# Supplementary material for: The Persistence of Facultative Parthenogenesis in Drosophila albomicans
Source: PLoS One. 2014 Nov 21;9(11):e113275. doi: 10.1371/journal.pone.0113275 (PMC4240631; doi:10.1371/journal.pone.0113275)
Supplement: Figure S1 — No evidence for Wolbachia infection in the parthenogenetic KKU119 of Drosophila albomicans. Wolbachia-specific primers, W-Specf and W-Specr [a], were used to amplify the 16S rRNA gene. To ensure the DNA quality, the primers, tLEU and tLYS [b], were used to amplify the mitochondrial COII gene. The D. ananassae strain 14024-0371.13 containing Wolbachia nuclear insert [c] was used as the positive control. Different numbers denote different KKU119 females. M: 100 bp DNA ladder. PC: positive control. NC: negative control. a. Werren JH, Windsor DM (2000) Wolbachia infection frequencies in insects: evidence of a global equilibrium? Proc R Sci Lond B 267: 1277-1285. b. Simon C, Frati F, Beckenbach A, Crespi B, Liu H, Floors P (1994) Evolution, weighting, and phylogenetic utility of mitochondrial gene sequences and a compilation of conserved polymerase chain reaction primers. Ann Entomol Soc Am 87: 651-701. c. Dunning Hotopp JC, Clark ME, Oliveira DCSG, Foster JM, Fischer P, et al. (2007) Widespread lateral gene transfer from intracellular bacteria to multicellular eukaryotes. Science 1753-1756. (DOCX) [file pone.0113275.s001.docx]

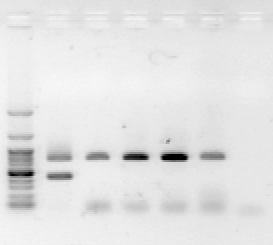


*D. albomicans* KKU119

NC

1

2

3

4

*COII*

16S rRNA

M

PC

500 bp

1000 bp

Figure S1. No evidence for *Wolbachia* infection in the parthenogenetic KKU119 of *Drosophila albomicans. Wolbachia*-specific primers, *W-Specf* and *W-Specr* [a], were used to amplify the *16S rRNA* gene. To ensure the DNA quality, the primers, *tLEU* and *tLYS* [b], were used to amplify the mitochondrial *COII* gene. The *D. ananassae* strain 14024-0371.13 containing *Wolbachia* nuclear insert [c] was used as the positive control. Different numbers denote different KKU119 females. M: 100 bp DNA ladder. PC: positive control. NC: negative control.

a. Werren JH, Windsor DM (2000) *Wolbachia* infection frequencies in insects: evidence of a global equilibrium? Proc R Sci Lond B 267: 1277-1285.

b. Simon C, Frati F, Beckenbach A, Crespi B, Liu H, Floors P (1994) Evolution, weighting, and phylogenetic utility of mitochondrial gene sequences and a compilation of conserved polymerase chain reaction primers. Ann Entomol Soc Am 87: 651-701.

c. Dunning Hotopp JC, Clark ME, Oliveira DCSG, Foster JM, Fischer P, et al. (2007) Widespread lateral gene transfer from intracellular bacteria to multicellular eukaryotes. Science 1753-1756.
