# Supplementary material for: The Persistence of Facultative Parthenogenesis in Drosophila albomicans
Source: PLoS One. 2014 Nov 21;9(11):e113275. doi: 10.1371/journal.pone.0113275 (PMC4240631; doi:10.1371/journal.pone.0113275)
Supplement: Table S1 — Diploidization mechanisms of F1 female Drosophila albomicans with more than one offspring. (DOCX) [file pone.0113275.s002.docx]

Table S1. Diploidization mechanisms of F_1_ female *Drosophila albomicans* with more than one offspring.

| Cross set | No. total offspring | Gamete duplication | Fusion* | Uncertain | No. virgin F_1_ |
| --- | --- | --- | --- | --- | --- |
| KKU119 X #55.1 | 2 |  |  | 2 | 2 |
|  | 2 | 2 |  |  | 3 |
|  | 2 | 1 |  | 1 | 4 |
|  | 2 | 1 | 1 |  | 1^†^ |
|  | 3 | 1 |  | 2 | 1 |
|  | 3 | 3 |  |  | 1 |
|  | 4 | 1 |  | 3 | 1 |
|  | 4 | 2 |  | 2 | 4 |
|  | 4 | 1 | 1 | 2 | 1^†^ |
|  | 4 | 2 | 1 | 1 | 1^†^ |
|  |  |  |  |  |  |
| KKU119 X #163.5-IL | 2 |  |  | 2 | 4 |
|  | 2 | 2 |  |  | 3 |
|  | 2 | 1 |  | 1 | 7 |
|  | 2 | 1 | 1 |  | 1^†^ |
|  | 3 |  |  | 3 | 2 |
|  | 3 |  | 1 | 2 | 1 |
|  | 3 | 1 |  | 2 | 1 |
|  | 3 | 2 |  | 1 | 1 |
|  | 3 | 1 | 1 | 1 | 1^†^ |
|  | 4 | 1 |  | 3 | 1 |
|  | 5 |  |  | 5 | 1 |

*Fusion: terminal fusion or central fusion

^†^Females produced parthenogenetic offspring by multiple diploidization mechanisms.
