# Supplementary material for: The Persistence of Facultative Parthenogenesis in Drosophila albomicans
Source: PLoS One. 2014 Nov 21;9(11):e113275. doi: 10.1371/journal.pone.0113275 (PMC4240631; doi:10.1371/journal.pone.0113275)
Supplement: Table S2 — The average cumulative number of eggs laid by 4-day-old virgin and mated females of Drosophila albomicans. (DOCX) [file pone.0113275.s003.docx]

Table S2. The average cumulative number of eggs laid by 4-day-old virgin and mated females of *Drosophila albomicans*.

|  | Average number of eggs^a^ | |
| --- | --- | --- |
| Strain | Virgin female | Mated female |
| #55.1 | 36 ± 26.1 (13)^b^ | 54 ± 12.8 (13) |
| KKU119 | 38 ± 18.6 (15) | 66 ± 25.4 (6) |

^a^ Average cumulative number of eggs laid per female in four days.

^b^ Sample sizes in parentheses.
